# Role of parental educational level as psychosocial factor in a sample of inpatients with anorexia nervosa and bulimia nervosa

**DOI:** 10.3389/fpsyg.2024.1408695

**Published:** 2024-05-17

**Authors:** Francesco Bevione, Matteo Martini, Paola Longo, Federica Toppino, Alessandro Musetti, Laura Amodeo, Giovanni Abbate-Daga, Matteo Panero

**Affiliations:** ^1^Eating Disorders Unit, Department of Neuroscience "Rita Levi Montalcini", University of Turin, Turin, Italy; ^2^Department of Humanities, Social Sciences and Cultural Industries, University of Parma, Parma, Italy

**Keywords:** eating disorders, eating psychopathology, anorexia nervosa, psychosocial risk factors, parental educational level

## Abstract

**Introduction:**

Evidence on parental educational level (PEL) as a risk factor for Eating Disorders (EDs) is mixed, and no study has assessed its role in relation to the compliance and outcomes of treatments in EDs. Further, no study differentiated from the educational level of mothers and fathers, nor considered the possible mediation of perfectionism in fostering EDs.

**Methods:**

A clinical sample of 242 first-ever admitted inpatients with EDs provided information on PEL and completed the following questionnaires: the Eating Disorder Examination Questionnaire (EDE-Q) and the Frost Multidimensional Perfectionism Scale (F-MPS). Clinicians also provided information on the Hamilton Rating Scale for Anxiety (HAM-A) and the Hamilton Rating Scale for Depression (HAM-D) for each participant.

**Results:**

Individuals with high PEL (whether mothers, fathers, or both parents) showed significantly higher scores on depressive symptoms and lower on parental criticism, were younger, had an earlier age of onset, had fewer years of illness, more were students and employed, and fewer had offspring. Individuals with fathers or both parents with high educational levels suffered more from Anorexia Nervosa rather than Bulimia Nervosa, had a longer length of stay during the current hospitalization, had less dietary restraint, and had higher personal standards. Individuals with mothers with high educational levels showed a lower rate of previous substance or alcohol addiction. Personal standards partially mediated the relationship between higher PEL and lower dietary restraint.

**Discussion:**

PEL emerged to be a twofold psychosocial risk factor, being associated with higher depressive symptoms and a longer length of stay, but also with a shorter duration of illness and better scholar and working involvement. Higher PEL was related to higher personal standards but not to global perfectionism. Patterns of eating psychopathology emerged based on the high PEL of mothers or fathers.

## 1 Introduction

Educational level (EL) refers to the highest level of education an individual has completed. Parental educational attainment or parental educational level (PEL) is thought to influence in many ways children’s development (Kraaykamp and van Eijck, 2010), behavior (Horoz et al., 2022), academic achievement (Schlechter and Milevsky, 2010), and physical health (Naess et al., 2018; Balaj et al., 2021). On the one hand, highly educated parents are believed to have better time spent with their children in terms of quality and quantity and provide them with better socioeconomic conditions and opportunities (Reiss et al., 2019). Also, a higher parental educational level was associated with a less permissive and less authoritarian parenting style (Hadjicharalambous and Dimitriou, 2020), higher assistance in their children’s school performance (Hadjicharalambous and Dimitriou, 2020), and greater use of the contingent shift principle rather than fixed failure feedback (Carr and Pike, 2012). Through the mediation of early maladaptive schemas, a link between perceived parent styles and eating disorder symptoms emerged in recent studies (Khosravi et al., 2023; Joshua et al., 2024). On the other hand, higher parents’ academic attainment may cause adolescent offspring to have increased academic distress perception and worsen well-being (Jang et al., 2020). In some studies, highly educated mothers have been described as perceiving more distress concerning motherhood, with worse parenting styles (Andersson and Hildingsson, 2016). Higher PEL was also described as a risk factor for schizophrenia (Byrne et al., 2004). Conversely, a lower PEL was associated with increased psychiatric disorders prevalence among offspring. Two longitudinal studies of 2,810 participants of the German KiGGS study (Meyrose et al., 2018) and of 10,257 participants from the Norwegian Patient Registry (Bøe et al., 2021) evidenced that children of mothers with low education have a significantly higher risk of mental health issues compared to those of mothers with high education, especially during the first phase of childhood. Three extensive longitudinal studies evidenced that children of parents with low PEL have a significantly increased risk of depression (Quesnel-Vallée and Taylor, 2012; Park et al., 2013; Fakhrunnisak and Patria, 2022). Results from the Canadian National Population Health Survey (Park et al., 2013) showed that the offspring of mothers with less than secondary school education had twofold higher odds of major depressive episodes during adulthood. Results from the Indonesia Family Life Survey (Fakhrunnisak and Patria, 2022) replicated those findings, evidencing slight differences depending on the educational level of mothers or fathers and the sex of children. A recent meta-analysis (Xiang et al., 2023) confirmed this evidence, displaying that overall parental education level was negatively correlated with adolescent depressive symptoms with a dose–response relationship. Low parents’ educational level was also associated with ADHD prevalence (Torvik et al., 2020) and suicidal behaviors (Chen et al., 2022) in children.

For what regards eating disorders (EDs), evidence is mixed. In detail, six extensive longitudinal studies evidenced an increased prevalence of EDs in families with higher PEL (Ahrén-Moonga et al., 2009; Ahrén et al., 2013; Goodman et al., 2014; Bould et al., 2016; Sundquist et al., 2016; Koch et al., 2022). Parents’ educational attainment as an associated factor was independent of parents’ social class, parental income (Ahrén et al., 2013; Goodman et al., 2014; Bould et al., 2016), also regarded grandparents’ educational level (Ahrén-Moonga et al., 2009; Goodman et al., 2014), selectively female offspring (Ahrén et al., 2013; Sundquist et al., 2016), and was related to health outcomes (O’Brien et al., 2017). However, another large longitudinal study of 11,721 children from the ABCD Study showed no differences in lifetime ED prevalence based on PEL (Sanzari et al., 2023). Other studies also repeated these findings of the non-significance of PEL on ED prevalence among children (Roberts et al., 2007; Pilecki et al., 2016; Udo and Grilo, 2018). This heterogeneity among findings suggests that the relationship between PEL and EDs may be complex and depending on several reasons.

Educational attainment in individuals themselves – which is influenced by PEL (Schlechter and Milevsky, 2010) – is debated as a risk factor. Most of the evidence proposes school performance as a promoting factor for EDs (Ahrén-Moonga et al., 2009; Sundquist et al., 2016; Claydon and Zullig, 2020; Schilder et al., 2021; Barakat et al., 2023; Nielsen and Vilmar, 2024), but some studies have evidenced a negative association between academic grades and prevalence of EDs (Maxwell et al., 2011; Adelantado-Renau et al., 2018). A systematic review of 149 full-text articles also concluded that education level is inconsistent as a risk factor for EDs (Mitchison and Hay, 2014).

The contrasting findings could be partially explained, considering that academic performance and parental push to excel in studying could be experienced in different ways by individuals. From this perspective, scholar attainment and parents’ education should be considered not only as possible risk factors but also in relation to their psychological consequences on individuals. Perfectionism has been proposed to be, in some cases, the link factor between high educational achievement and the risk of EDs (Schilder et al., 2021), given its well-established action on fostering eating psychopathology (Dahlenburg et al., 2019; Longo et al., 2024). A meta-analysis of 23 studies for 3,561 participants (Dahlenburg et al., 2019) showed that maladaptive and adaptive perfectionism (i.e., perfectionism consisting of high personal standards and aims) is increased in AN patients compared to healthy controls. This finding was also confirmed by a recent study (Longo et al., 2022) through a cluster analysis based on the Frost Multidimensional Perfectionism Scale (F-MPS), which outlined how the “high-perfectionism” subgroup was significantly more extensive compared to the group of healthy controls with this feature. The same research also evidenced that perfectionism scores were linked to more severe eating psychopathology – both in patients and controls – and to heavier depression and anxiety levels. Also, a network analysis (Martini et al., 2021) identified essential associations among perfectionism, interoceptive sensibility, and eating symptomatology. Among other possible links between higher PEL and ED psychopathology, psychological characteristics associated with high academic studies include introversion, locus of control, and engagement (Hakimi et al., 2011; Muca et al., 2023). Further, maintaining school performance despite weight loss has been found to produce empowerment, consequently becoming a factor of resistance to treatment (Abbate-Daga et al., 2013). Conversely, impairment of school marks during illness is perceived as an egodystonic factor and could reinforce compliance to treatments (Gregertsen et al., 2017).

The experience of academic pressure on individuals is a complex issue. Many variables could mediate the connection between PEL and eating psychopathology among offspring, and the same educational situation might foster different behaviors in different individuals. A deeper and more precise analysis of this relation would allow more knowledge of the pathways that lead to eating psychopathology and possibly open the way for more tailored possibilities for treatments.

Particularly interesting in the perspective of care would be to analyze the association of PEL with treatment outcomes. In this sense, a crucial event in the course of illness is the first-ever hospitalization since individuals experience acute and substantial difficulties. Further, clinicians should provide a prognosis during hospitalization to define the most appropriate path of care for individuals (Monteleone et al., 2023). As more variables are considered in the process of prognosis formulation, the course of treatment post-discharge will be better tailored. To our knowledge, no study has assessed the role of PEL on the compliance and outcome of treatment in EDs. Furthermore, no evidence is available concerning the association of PEL with ED outcomes during hospitalization, and no study in the literature has explored the role of PEL concerning ED psychopathology distinguished by the educational level of mothers and fathers. These are significant gaps in the literature that incited us to examine the role of PEL as a psychosocial factor associated with EDs, particularly contextualized to the first-ever hospitalization.

Given these premises, we intended to conduct an exploratory analysis to assess how PEL is associated with eating and general psychopathology differences in individuals with Anorexia Nervosa (AN) and Bulimia Nervosa (BN). To do that, we aimed to compare ED individuals with high and low PEL for what involves clinical data and psychometric questionnaires, separately considering mothers’ educational level (MPEL), fathers’ educational level (FPEL), and both parents’ educational level (PPEL). From this perspective, we were especially interested in the dimension of perfectionism, given the described role of perfectionism both in EDs and at the educational level.

Based on the evidence available in the literature, we expected that higher PPEL was related to AN but not BN symptomatology and lower PPEL to anxiety and depression (Quesnel-Vallée and Taylor, 2012; Park et al., 2013; Sundquist et al., 2016; Fakhrunnisak and Patria, 2022; Koch et al., 2022). We did not expect any finding based on selective MPEL and FPEL since no evidence was available in the literature.

## 2 Methods

### 2.1 Participants

Two hundred and forty-two first-ever admitted inpatients with AN or BN at the Eating Disorders Center of the “Città della Salute e della Scienza” hospital of the University of Turin, Italy, were recruited. This specialized psychiatric unit for ED recruits individuals during the acute stage of illness. This unit is the only in the Piedmont region to do so, thus recruiting individuals from different and varied care pathways. All patients were diagnosed with EDs according to DSM-5 (American Psychiatric Association, 2013) by an experienced psychiatrist following the Structured Clinical Interview for DSM-5 (First, 2015). The following inclusion criteria were respected: (a) age range: 18–35 years old; (b) diagnosis of EDs according to the Structured Clinical Interview for DSM-5 (First, 2015). No exclusion criteria were set.

We chose to investigate young adults since we aimed to study the effects of PPEL during a time of life in which the influence of parental characteristics may be considered relevant. The age span of 18–35 for young adults has been chosen by comparison with other work on the same issue (Franssen et al., 2020). We did not include underage patients since the sample was collected in an adult psychiatric service.

No participant refused to fill in the questionnaires. All individuals voluntarily agreed to get involved in our study, declaring it through written informed consent according to the Ethical Committee of our Institution, which also approved the present study under registration number 00295/2021 of 9/6/2021.

### 2.2 Procedure and measures

Trained nursing personnel measured individuals in height and weight. Subsequently, participants were interviewed by an experienced psychiatrist who collected clinical and demographic data. Specifically, for each patient, we collected information regarding age, gender, marital status, education level, illness onset, caloric intake, working and studying status, having siblings and children, previous failure of psychotherapeutic or pharmacological treatments (as reported by patients), self-harm, suicidal behavior, sexual abuse, current and previous substance or alcohol abuse, and family history of psychiatric and eating disorders. Each psychiatrist filled in the Hamilton Rating Scale for Anxiety (HAM-A; Hamilton, 1959) and the Hamilton Rating Scale for Depression (HAM-D; Hamilton, 1960), to have clinician-rated measures of anxious and depressive symptomatology, respectively. At the end of the hospitalization, nursing personnel measured individuals’ weight to derive the difference in BMI between hospital admission and discharge (ΔBMI). Information regarding PEL was also collected. The academic course in Italy is divided into steps. Specifically, elementary school (5 years of education), middle school (in total 8 years of education), first three-year period of secondary school (end of the mandatory schooling – in total 11 years of education), secondary school (in total 13 years of education), and academic degree (in total > thirteen years of education). For data collection, it was not possible to obtain information on the parents’ academic level attainment (i.e., elementary school, middle school, high school diploma, academic degree), hence, we used the parents’ years of schooling reported by participants.

All participants were asked to complete the following self-report questionnaires:

- Eating Disorder Examination Questionnaire (EDE-Q; Fairburn and Beglin, 1994). This questionnaire assess attitudes and behaviors of eating disorders during the previous 28 days. It is composed of 28 items with four subscales: “dietary restraint,” “eating concerns,” “weight concerns,” “shape concerns,” and a total score. The Italian version of the questionnaire provided a high internal consistency, as suggested by a Cronbach alpha value >0.90 (Calugi et al., 2017).
- Frost Multidimensional Perfectionism Scale (F-MPS; Frost et al., 1990). This questionnaire was included to explore the main aspects of perfectionism. It is made up of 35 items on a five-point scale ranging from 1 (total disagreement) to 5 (complete agreement), and its answers constitute seven subscales: “concern over mistakes,” “personal standards,” “parental expectations,” “parental criticism,” “doubts about actions,” “organization,” and total score. Higher scores indicate more pronounced perfectionism traits. The Italian version of this tool proved to have good internal consistency, with a Cronbach alpha value >0.75 (Lombardo, 2008).
- The clinicians were trained to assess anxiety and depression using the Italian translation of the following tools:
  - Hamilton Rating Scale for Anxiety (HAM-A; Hamilton, 1959). This clinician-rated scale was included to measure the severity of anxiety symptoms. It provides measures of “overall anxiety,” “psychic anxiety,” and “somatic anxiety.”
  - Hamilton Rating Scale for Depression (HAM-D; Hamilton, 1960). This clinician-rated scale was utilized to measure the severity of depressive symptoms in individuals. The score indicates “possible” (10–15), “light” (16–25), “moderate” (26–28), and “severe” (>28) depression. The scale proved to be effective in separating depressive and healthy individuals in an Italian sample (Fava et al., 1982).

### 2.3 Statistical analysis

All statistical analyses were performed utilizing IBM SPSS 28.0 Statistics Software (SPSS).

To compare individuals based on the PEL, we referred to the parents’ years of education as described above. We decided to group individuals with parents of PEL <13 years of education and PEL ≥13 years of education. This partition was set up for two reasons. First, in Italian education, ≥13 years of education corresponds to having an academic degree, and so having accomplished a high-school diploma. We believed the high-school diploma was generally the most significant step across the educational path of an individual. Second, it provided us with two subgroups of individuals with similar numerousness, thus allowing us statistical comparisons between them. Specifically, we compared individuals with mothers of <13 and ≥ 13 years of education (MPEL), individuals with fathers of <13 and ≥ 13 years of education (FPEL), and individuals with at least one parent of PEL <13 years of education with individuals with both parents of PEL ≥13 years of education (PPEL). Hence, our comparison was between parents with the education of “high-school diploma or above” and “below high-school diploma.” The detailed description of PEL in fathers and mothers is described in Supplementary Table S1.

These comparisons were conducted with a chi-squared test for nominal variables and a t-test for continuous variables. Furthermore a chi-squared test was conducted between MPEL and FPEL.

Secondly, we ran a logistic regression in which PPEL was the dependent variable. The independent variables were those that significantly differed (at least one among MPEL, FPEL, and PPEL) at the previous analysis (i.e., age, age of onset, years of illness, being student, being employed, HAM-D, EDE-Q dietary restraint, F-MPS personal standard, F-MPS parental criticism). Cox and Snell *R*^2^ was calculated.

Since our analysis was exploratory and there was no omnibus null hypothesis about which all the tests speak collectively, according to Garcia-Perez’s indications (García-Pérez, 2023) we decided to avoid a correction for multiple testing.

Finally, two mediation models of the relationship between PEL and EDE-Q restraint were employed to assess the possible mediating effects of participants’ personal standards and perceived parental criticism. We utilized the corresponding subscores on the F-MPS. These variables were chosen because they agreed with the hypothesis of perfectionism as a mediator and were significant in the previous analysis.

## 3 Results

The clinical sample was composed of 242 individuals. All individuals provided information on the mothers’ and 238 on the fathers’ educational level. Hundred and fifty-two individuals were diagnosed with the restricting AN (AN-R) subtype (62.8%), 64 with the binge-purging subtype (AN-BP) (26.4%), and 26 with BN (10.7%) (see Supplementary Table S2). The AN sample was characterized by severe underweight and a long duration of illness. Mothers were 28.2% with <13 years and 64.4% with ≥13 years of education. Fathers were 32.2% with <13 years and 54.6% with ≥13 years of education. Individuals with high PEL (MPEL, FPEL and PPEL) were more frequently single and less frequently married/cohabitant as described in Supplementary Table S3. The distribution of PEL in parents was asymmetrical. The vast majority (87.6%) of parental pairs were homogeneous for PEL (i.e., with both parents having a high or low PEL) as described in Supplementary Table S4. The distribution of PEL and the individuals’ marital status are described in Supplementary Tables S1, S3.

### 3.1 Differences between ED individuals with high and low PEL

Differences between ED individuals with high and low PEL are described in Table 1.

**Table 1:** Differences between EDs individuals with parents with high and low PEL.

ED Inpatients	Mothers of ≥13 years of education (*n* = 156) Mean (SD) or *N* (%)	Mothers of <13 years of education (*n* = 86) Mean (SD) or *N* (%)	χ^2^	*F*	*p*	Fathers of ≥13 years of education (*n* = 135) Mean (SD) or *N* (%)	Fathers of <13 years of education (n = 103) Mean (SD) or *N* (%)	χ^2^	*F*	*p*	Both parents of ≥13 years of education (*n* = 124) Mean (SD) or *N* (%)	At least one parent of <13 years of education (*n* = 114) Mean (SD) or *N* (%)	*χ* ^2^	F	p
AN	141 (90.4%)	75 (87.2%)	0.583	–	0.445	**128 (94.8%)**	**86 (83.5%)**	**8.256**	**–**	**0.004**	**118 (95.2%)**	**96 (84.2%)**	**8.581**	**-**	**0.003**
BN	15 (9.6%)	11 (12.8%)	0.583	–	0.445	**7 (5.2%)**	**17 (16.5%)**	**8.256**	**–**	**0.004**	**6 (4.8%)**	**18 (15.8%)**	**8.581**	**-**	**0.003**
Age	**22.0 (7.1)**	**26.5 (10.2)**	**–**	**15.789**	**<0.001**	**21.4 (6.4)**	**26.3 (10.0)**	**–**	**21.329**	**<0.001**	**21.5 (6.6)**	**26.0 (9.8)**	**–**	**16.742**	**<0.001**
Age of onset	**16.8 (4.2)**	**18.3 (6.1)**	**–**	**6.283**	**0.013**	**16.5 (3.1)**	**18.1 (6.1)**	**–**	**16.822**	**<0.001**	**16.6 (3.2)**	**18.5 (6.3)**	**–**	**18.067**	**<0.001**
Years of Illness	**5.4 (6.4)**	**8.1 (9.5)**	**–**	**12.581**	**0.008**	**8.1 (9.3)**	**5.0 (6.2)**	**–**	**15.488**	**0.002**	**5.0 (6.3)**	**7.8 (9.0)**	**–**	**10.905**	**0.006**
Having siblings	115 (73.7%)	71 (82.6%)	2.197	–	0.138	97 (71.5%)	84 (81.6%)	1.855	–	0.173	92 (74.2%)	94 (82.5%)	1.888	-	0.169
Having offspring	**0 (0.0%)**	**6 (7.0%)**	**11.160**	**–**	**<0.001**	**0 (0.0%)**	**6 (5.8%)**	**7.891**	**–**	**0.005**	**0 (0.0%)**	**6 (5.3%)**	**6.683**	**-**	**0.010**
Being student	**112 (71.8%)**	**30 (34.9%)**	**31.151**	**–**	**<0.001**	**94 (69.6%)**	**42 (40.8%)**	**21.982**	**–**	**<0.001**	**92 (74.2%)**	**50 (43.9%)**	**22.287**	**-**	**<0.001**
Being employed	**120 (76.9%)**	**38 (44.2%)**	**25.293**	**–**	**<0.001**	**99 (73.3%)**	**53 (51.5%)**	**14.733**	**–**	**<0.001**	**97 (78.2%)**	**61 (53.5%)**	**15.262**	**-**	**<0.001**
Educational level in years	13.4 (2.7)	13.3 (2.8)	–	0.032	0.651	13.3 (2.7)	13.4 (2.7)	–	0.097	0.903	13.3 (2.7)	13.4 (2.7)	–	0.133	0.808
Previous failure of psychotherapy	72 (46.1%)	32 (37.2%)	2.304	–	0.129	63 (46.7%)	39 (37.9%)	2.596	–	0.107	61 (49.2%)	43 (37.7%)	3.597	-	0.058
Previous failure of pharmacotherapy	49 (31.4%)	27 (31.4%)	0.040	–	0.841	45 (33.3%)	31 (30.1%)	0.648	–	0.421	42 (33.9%)	34 (29.8%)	0.715	-	0.398
Presence of self-harm behaviors	27 (17.3%)	9 (10.5%)	2.162	–	0.141	19 (14.1%)	15 (14.6%)	<0.001	–	0.991	19 (15.3%)	17 (14.9%)	0.021	-	0.885
Presence of suicidal behaviors	16 (10.3%)	12 (13.9%)	0.680	–	0.410	12 (8.9%)	13 (12.6%)	0.690	–	0.406	11 (8.9%)	17 (14.9%)	1.946	-	0.163
Number of suicidal attempts in anamnesis	0.2 (0.7)	0.4 (0.8)	–	3.157	0.274	0.2 (0.7)	0.3 (0.9)	–	2.994	0.316	0.2 (0.6)	0.4 (0.9)	–	7.260	0.127
History of sexual abuse	14 (9.0%)	9 (10.5%)	0.120	–	0.729	8 (5.9%)	13 (12.6%)	2.996	–	0.083	8 (6.4%)	15 (13.2%)	3.051	-	0.081
Current substance or alcohol abuse	6 (3.8%)	6 (7.0%)	1.128	–	0.288	7 (5.2%)	5 (4.8%)	0.028	–	0.866	6 (4.8%)	6 (5.3%)	0.018	-	0.894
Previous substance or alcohol addiction	**5 (3.2%)**	**9 (10.5%)**	**5.468**	**–**	**0.019**	6 (4.4%)	8 (7.8%)	1.069	–	0.301	5 (4.0%)	9 (7.9%)	1.565	-	0.211
Family history of psychiatric diseases	62 (39.7%)	25 (29.1%)	2.663	–	0.103	46 (34.1%)	36 (34.9%)	0.005	–	0.945	44 (35.5%)	43 (37.7%)	0.124	-	0.724
Family history of Eating Disorders	30 (19.2%)	9 (10.5%)	3.223	–	0.073	23 (17.0%)	14 (13.6%)	0.681	–	0.409	23 (18.5%)	16 (14.0%)	0.941	-	0.332
HAM-A	19.9 (7.0)	18.6 (6.2)	–	1.363	0.204	19.7 (6.4)	18.9 (7.0)	–	0.060	0.360	19.8 (6.5)	19.0 (7.0)	–	0.024	0.392
HAM-D	**19.4 (7.7)**	**16.5 (5.8)**	**–**	**5.079**	**0.004**	**19.3 (7.5)**	**17.2 (6.8)**	**–**	**1.960**	**0.035**	**19.4 (7.5)**	**17.3 (6.7)**	**–**	**2.590**	**0.036**
EDE-Q, dietary restraint	3.2 (2.0)	3.8 (1.9)	–	1.091	0.074	**3.1 (2.0)**	**3.9 (1.9)**	**–**	**3.577**	**0.008**	**3.1 (2.0)**	**3.8 (2.0)**	**–**	**1.130**	**0.029**
EDE-Q, eating concerns	3.1 (1.6)	3.3 (1.7)	–	0.108	0.365	3.0 (1.6)	3.4 (1.7)	**–**	0.501	0.080	3.0 (1.6)	3.3 (1.7)	–	0.913	0.145
EDE-Q, shape concerns	4.2 (1.7)	4.4 (1.6)	–	0.888	0.476	4.2 (1.7)	4.5 (1.5)	**–**	4.125	0.112	4.2 (1.7)	4.5 (1.5)	–	2.739	0.183
EDE-Q, weight concerns	3.8 (1.9)	3.8 (1.8)	–	0.400	0.948	3.7 (1.9)	3.9 (1.7)	**–**	2.068	0.393	3.7 (1.9)	3.8 (1.8)	–	0.980	0.593
EDE-Q, global score	3.6 (1.7)	3.8 (1.6)	–	0.911	0.352	3.5 (1.7)	3.9 (1.5)	**–**	2.796	0.085	3.5 (1.7)	3.8 (1.6)	**–**	1.263	0.173
F**–**MPS, concern over mistakes	30.8 (10.1)	31.6 (9.5)	–	1.176	0.557	30.8 (9.6)	31.5 (10.2)	**–**	0.038	0.602	30.9 (9.7)	31.3 (10.2)	**–**	0.007	0.728
F**–**MPS, personal standards	25.3 (6.7)	24.1 (6.5)	–	0.298	0.192	**25.9 (6.4)**	**23.8 (6.7)**	**–**	**0.050**	**0.023**	**25.9 (6.4)**	**23.8 (6.7)**	**–**	**0.156**	**0.019**
F-MPS, parental expectations	10.8 (5.3)	11.1 (5.4)	–	0.163	0.734	10.7 (5.2)	11.4 (5.5)	–	0.965	0.354	10.9 (5.3)	10.9 (5.4)	–	0.267	0.965
F-MPS, parental criticism	**9.5 (4.1)**	**10.9 (4.2)**	**–**	**0.120**	**0.014**	**9.3 (4.1)**	**11.0 (4.2)**	**–**	**0.185**	**0.004**	**9.3 (4.1)**	**10.6 (4.2)**	**–**	**0.010**	**0.029**
F-MPS, doubts about actions	13.0 (3.9)	13.8 (3.9)	–	0.352	0.170	12.9 (3.9)	13.8 (4.0)	–	0.028	0.112	13.0 (3.9)	13.6 (4.0)	–	0.002	0.223
F-MPS, organization	23.9 (5.4)	24.0 (4.8)	–	0.778	0.917	24.2 (5.2)	23.6 (5.3)	–	0.068	0.411	24.2 (5.2)	23.6 (5.2)	–	0.011	0.429
F-MPS, global score	113.0 (24.7)	114.9 (24.1)	–	0.716	0.597	113.9 (23.2)	114.2 (26.3)	–	0.214	0.916	114.2 (23.4)	113.0 (25.7)	–	0.021	0.719
BMI	15.0 (3.0)	15.0 (3.0)	–	0.470	0.976	14.7 (2.6)	15.3 (3.3)	–	1.289	0.149	14.8 (2.6)	15.3 (3.3)	–	2.303	0.153
Caloric Intake (kcal/day)	756.3 (421.8)	688.4 (348.4)	–	4.742	0.226	755.1 (414.7)	695.0 (359.5)	–	3.085	0.267	756.6 (417.3)	705.6 (375.6)	–	2.043	0.343
Length of stay during current hospitalization (in days)	35.0 (18.7)	31.5 (16.3)	–	2.125	0.157	**36.7 (19.7)**	**30.4 (15.3)**	**–**	**4.590**	**0.009**	**36.8 (20.0)**	**30.5 (14.8)**	**–**	**5.681**	**0.007**
Hospital discharge against physicians’ decision during the current hospitalization	4 (2.6%)	1 (1.2%)	0.467	–	0.495	2 (1.5%)	3 (2.9%)	0.567	–	0.452	2 (16.1%)	3 (2.6%)	0.326	-	0.568
ΔBMI	0.6 (0.9)	0.5 (0.7)	–	1.732	0.634	0.6 (0.9)	0.5 (0.7)	–	1.662	0.260	0.6 (0.9)	0.5 (0.8)	–	0.153	0.491
ΔBMI in the AN individuals’ subgroup	0.67 (0.91)	0.59 (0.69)	–	1.695	0.194	0.64 (0.87)	0.58 (0.65)	–	2.393	0.123	0.64 (0.88)	0.59 (0.80)	–	0.283	0.595

EDs, eating disorders; AN, Anorexia Nervosa; BN, Bulimia Nervosa; SD, standard deviation; N, number of individuals; χ^2^, chi-squared value; F = F-value; *p*, *p*-value; BMI, body mass index; ΔBMI, variation of BMI during the current hospitalization; HAM-A, Hamilton Anxiety Rating Scale; HAM-D, Hamilton Depression Rating Scale; EDE-Q, Eating Disorder Examination Questionnaire; F-MPS, Frost Multidimensional Perfectionism Scale. *Statistical significance if *p* < 0.05. Bold values *p* < 0.05.

Individuals with high MPEL, high FPEL, and high PPEL were younger, had an earlier age of onset, had fewer years of illness, a lower probability of having children, and a higher likelihood of being a student and being a worker. Individuals with high MPEL, high FPEL, and high PPEL showed significantly higher HAM-D scores and lower F-MPS parental criticism scores.

Individuals with high MPEL showed a lower probability of previous substance or alcohol addiction, but this was not present for individuals with high FPEL and PPEL.

Individuals with high FPEL and PPEL, but not MPEL, displayed a significantly higher probability of suffering from AN rather than BN, a longer length of stay during hospitalization, lower scores on EDE-Q dietary restraint, and higher scores on F-MPS personal standards.

No significant differences based on PEL emerged for what concerns the probability of having siblings, educational level, failure of previous pharmacological or psychotherapeutic trials, self-harm and suicidal behaviors, history of suicidal attempts, history of sexual abuse, current substance or alcohol abuse, family history of psychiatric diseases or ED, HAM-A score, the eating concerns, shape concerns, weight concerns and global score of EDE-Q, the concern over mistakes, parental expectations, doubts about actions, organization and global score of F-MPS, the state and trait STAI scores, the BSQ score, the Rosenberg score, caloric intake, BMI, ΔBMI, ΔBMI in the AN individuals’ subgroup, and hospital discharge against physicians’ decision during the current hospitalization.

### 3.2 Linear regression of PEL in ED individuals

We performed a linear regression to derive what dimensions correlate with the high PPEL in ED parents. Results are detailed in Table 2.

**Table 2:** Linear regression model for having both parents with high level of education (≥13 years of education) in EDs individuals.

	Multivariate Regression				Properties of the Model		
Variables	Beta	SE	W	*p*	*R*	*χ* ^2^	*p*
F-MPS, personal standards	**0.080**	**0.030**	**6.956**	**0.008**	**0.264**	**54.461**	**<0.001**
EDE-Q, dietary restraint	**−0.276**	**0.106**	**6.753**	**0.009**			
Age	**−0.099**	**0.049**	**4.148**	**0.042**			
HAM-D	**0.054**	**0.027**	**4.089**	**0.043**			
F-MPS, parental criticism	−0.086	0.047	3.285	0.070			
Being student	0.786	0.475	2.737	0.098			
Being employed	0.558	0.427	1.709	0.191			
Years of illness	0.061	0.048	1.628	0.202			

EDs, eating disorders; SE, standard Error; W, Wald’s score; R, Cox and Snell *R*^2^ score; EDE-Q, eating disorder examination questionnaire; HAM-D, Hamilton depression rating scale; F-MPS, Frost multidimensional perfectionism scale. *Statistical significance if *p* < 0.05. Degrees of freedom = 8. Bold values *p* < 0.05.

The linear regression model showed Cox and Snell *R*^2^ score = 0.264, χ^2^ = 54.461, and *p* < 0.001. Significant PPEL-related factors emerged to be F-MPS “personal standards,” EDE-Q “dietary restraint,” age, and HAM-D.

**Figure 1:**
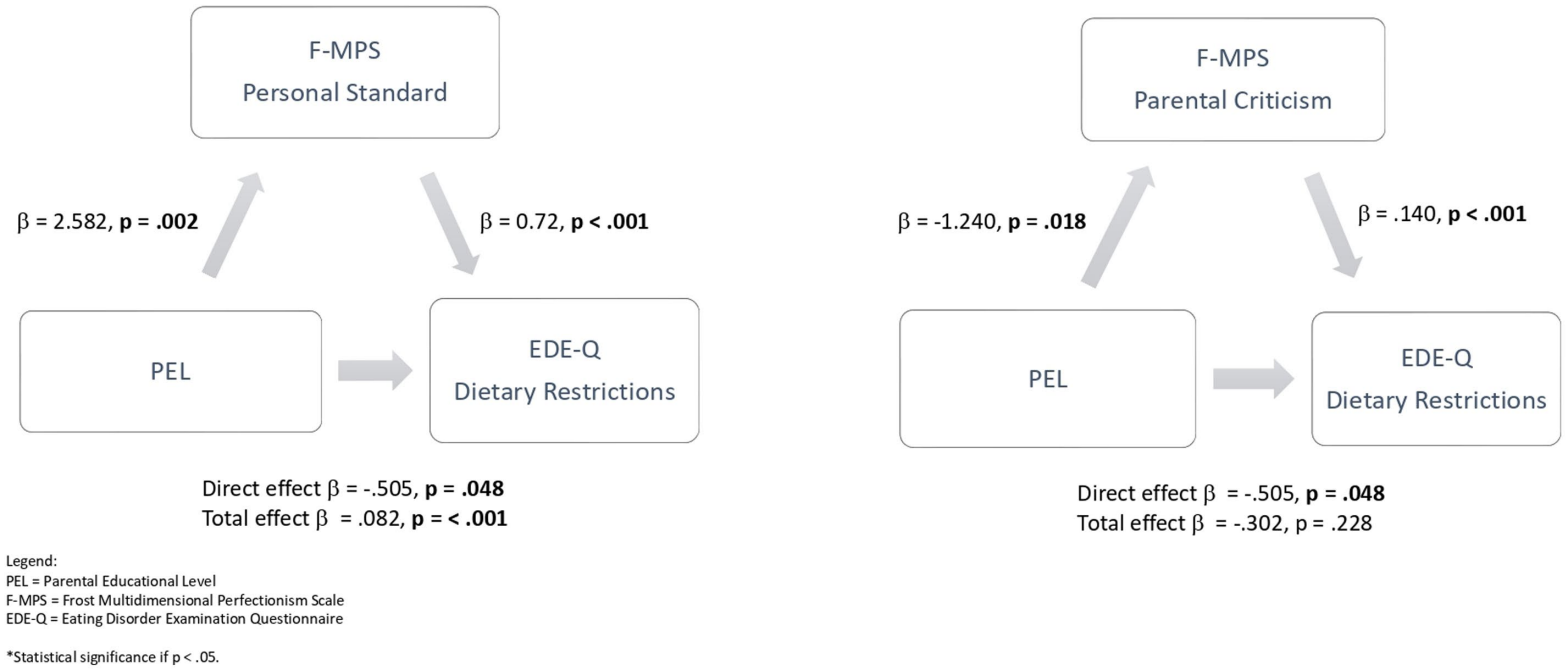
Mediation analysis of the relationship between PEL and dietary restrictions with personal standard as a mediator, and between PEL and dietary restrictions with parental criticism as a mediator. PEL, parental educational level; F-MPS, frost multidimensional perfectionism scale; EDE-Q, eating disorder examination questionnaire; *Statistical significance if *p* < 0.05. Bold values *p* < 0.05.

As shown in Figure 1 and Supplementary Tables S5, S6, the mediational model revealed personal standard’s significant partial mediation role in the relationships between PEL and dietary restraints. No mediation effect emerged regarding parental criticism in the same relationship. Even though no mediation effect of parental criticism emerged, the analysis confirmed a statistically significant association between parental criticism and dietary restraint (Supplementary Table S6).

## 4 Discussion

In our sample of first-ever admitted inpatients with EDs, individuals with high PEL (MPEL, FPEL, and PPEL) were younger, had an earlier age of onset, had fewer years of illness, fewer had offspring, and more were students and employed. Also, individuals with high PEL (MPEL, FPEL, and PPEL) showed significantly higher scores on depressive symptoms and lower scores on parental criticism. Individuals with fathers and both parents (FPEL and PPEL) but not mothers with high educational levels (MPEL) suffered more from AN rather than BN, had a longer length of stay (LoS) during the current hospitalization, and had less dietary restraint and higher personal standards. Individuals with mothers (MPEL) but not fathers and both parents with high educational levels (FPEL and PPEL) showed a significantly lower rate of previous substance or alcohol addiction.

No significant difference between the groups with high and low PEL (neither MPEL, FPEL, nor PPEL) was shown regarding BMI at admission, eating psychopathology (except for restraints), family history of psychiatric and eating disorders, and history of suffered events (i.e., sexual abuse, suicidal behavior, self-harm).

Hence, PEL *per se* did not emerge as a predictor of worse eating disorder symptoms presentation at admission. Still, higher PEL proved to be a psychosocial factor positively associated with comorbidities, such as the presence of depressive symptoms, which are a factor described as associated with longer LoS during inpatient treatments (Panero et al., 2021), and a history of substance abuse,. Surprisingly in our sample, higher PEL did not entail higher scores for patients’ perfectionism. This non-association can be explained by the fact that we measured perfectionism in the acute phase of the disease. In contrast, perfectionism may have played a role predominantly in the premorbid period.

The finding of higher depressive symptoms among ED individuals with high PEL was apparently in contrast with evidence showing a higher risk of depression among offspring of parents with low educational levels (Quesnel-Vallée and Taylor, 2012; Park et al., 2013; Fakhrunnisak and Patria, 2022). However, the cited studies measured the prevalence of clinically diagnosed cases of depression in the general population, while we assessed the depressive symptomatology among ED individuals. ED persons with high PEL might have a higher insight and awareness of their condition and possibly bear parental expectations that may produce a sense of failure. EDs have peculiar characteristics, and psychosocial risk factors like PEL can be different from other mental disorders.

FPEL and PPEL, but not MPEL, were associated with a significantly higher probability of suffering from AN than BN. These findings reinforced the hypothesis that AN is more frequently associated with higher PEL (Sundquist et al., 2016; Koch et al., 2022). Our study has the limitation of having involved mainly AN individuals (89.3%), and not having considered the individuals’ socioeconomic status. Consequently, we cannot exclude whether higher socio-economic status partially or totally mediated the relationship between higher PEL and AN. Nevertheless, whether mediated by socioeconomic status or not, our data propose that the presence of a highly educated father is associated with having AN rather than BN. Among fathers themselves, higher education might be associated with restrictive and orthorexic rather than binge eating and purging eating habits (McComb and Mills, 2019; Gkiouleka et al., 2022), which might influence siblings’ eating behavior.

The subscores of perfectionism dimensions on F-MPS indicated a lower perception of parental criticism among individuals with high PEL (MPEL, FPEL, and PPEL), but higher personal standards in individuals with high FPEL and PPEL. We can hypothesize that individuals may perceive highly educated parents not as strict and pressing but as encouraging. However, higher personal standards scores might entail parents internalized as hard-to-reach models, resulting in difficulties in perceiving themselves as effective and successful (Panero et al., 2022). These findings reinforce the need for complex models based on several variables. Perfectionism has been repeatedly associated with restricting eating behaviors (Dahlenburg et al., 2019; Martini et al., 2021; Longo et al., 2022, 2024). In our sample, global perfectionism was not significantly different between the groups with high and low PEL. However, features of perfectionism, such as high personal standards, were related to higher PEL. In our sample, individuals with high PEL were more involved in scholarly and working activities. On the one hand, this could be viewed as the result of more educational and career opportunities for the offspring of parents with high PEL. On the other hand, individuals with high PEL and higher personal functioning may also present higher individuals’ expectations that are linked to perfectionism (Longo et al., 2024). In the linear regression model, the personal standards dimension of perfectionism was shown to be the strongest factor for having both parents with high PEL (see Table 2).

Higher PEL was associated with lower dietary restraint (EDE-Q) among participants. This relationship had a partial mediation of personal standards, and no mediation emerged for parental criticism. In previous research, perfectionism has been proposed to be the link between high educational achievement and the risk of EDs (Schilder et al., 2021). This exploratory analysis described how the presence of high parental criticism may be the feature of perfectionism that mediated between PEL and eating symptoms, thus deepening the knowledge of the association between ED and perfectionism (Dahlenburg et al., 2019). As we described above, higher PEL was linked to a higher prevalence of AN than BN. However, in our sample of ED individuals, higher PEL was associated with lower restrictive symptoms. We must underline that we recruited ED individuals during their first-ever hospitalization, thus in a very acute stage. In this context, higher PEL might be associated with lower restrictive symptoms in the context of a clinical diagnosis of AN, thus playing a positive role.

High PEL was associated with a younger age, earlier onset, and fewer years of illness. The earlier age of onset was explainable in two ways. On the one hand, it could represent an earlier distress sign and negative prognostic factor. However, on the other hand, higher PEL may correspond to an increased and improved time parents spend with their children (Oreopoulos et al., 2006), thus to higher attention on children’s suffering and more economical and social possibilities of treatment. In this perspective, the younger age of onset might be due to an earlier detection of EDs cases. If the data are confirmed, higher PEL could be connected to earlier detection and intervention, representing a positive psychosocial factor associated with better outcomes in the long period (Treasure and Russell, 2011; Fukutomi et al., 2020; Austin et al., 2022).

Individuals with high PEL were more single and less engaged/married (see Supplementary Table S3). As we have seen, they were also more likely to be students and workers. These data could be at least partially due to an increased emphasis of families with high PEL on the importance of study and career, resulting in higher rates of schooling and employment, and a delay in romantic relationships.

Finally, individuals with fathers and both parents with high EL required a longer duration of the first-ever hospitalization, whereas no difference emerged concerning discharge against physicians’ decision. Since depression levels in a previous study were shown to increase the length of stay of hospitalization in AN (Panero et al., 2021), this association might be at least partially due to the higher depressive symptomatology among individuals with high PEL. In any case, clinicians must consider higher PEL as a psychosocial factor that could foster a lengthening of hospital treatments. This finding underscores the importance of considering the broader social context when developing treatment plans, and how therapeutic approaches could be tailored to accommodate the diverse needs of individuals from varying socioeconomic backgrounds (Marzola et al., 2022; Monteleone et al., 2023). Further, providing education and support to ED individuals and their families could enhance understanding of the condition and empower families to play a more active role in the treatment process (Couturier et al., 2010; Datta et al., 2023). This may involve offering resources such as psychoeducation, support groups, or counseling services (Treasure and Schmidt, 2013). In fact, in the Italian context of ED paths of care, multidisciplinary treatment models involving and including interventions on family members proved to lead to a better prognosis (Castellini et al., 2023). In this perspective, it would be all the more essential to identify moderators (like PEL).

## 5 Conclusion

Our study evidenced some interesting findings. First, PEL emerged as a twofold psychosocial factor. On the one hand, during hospitalization in an acute phase of the illness, in our sample of ED individuals, high PEL was associated with higher depressive symptoms and a longer length of stay. To achieve the same weight gain, patients with high PEL required more time and effort, and hence, more resources to be allocated. However, on the other hand, higher PEL was linked to a shorter duration of illness, and a higher scholar and working involvement. Second, higher PEL was not straightforwardly related to global perfectionism. Nevertheless, it was associated with higher personal standards, the strongest factor related to high PEL. Third, personal standards mediated the relationship between PEL and dietary restraint. Finally, the presence of a high educational level only in one parent presented some peculiar patterns. Specifically, suffering from AN rather than BN was associated with high paternal EL, and a history of substance abuse with low maternal EL. More research is required to identify peculiarities like these to differentiate factors such as paternal and maternal EL.

Some limitations of our study must be acknowledged. Firstly, we observed ED individuals during their first-ever hospitalization, hence during a very acute phase of the disorder. This offered us the possibility of a point of view different from what is available in literature. On the other hand, this implicated the bias of the possible existence of state effects that could be in addition to the trait ones. Second, the small size of the BN sample did not allow any sub-analysis based on diagnostic subtypes. Third, we utilized mainly self-report measures for what concerns psychological variables, and the parents’ years of schooling as a proxy for their education level. Further, it was not possible to have information on the parents’ marital status, which could act as a confounding factor. Fourth, the cross-sectional study design did not allow us to conclude any causal relation but only statistical associations between variables. Fifth, since the sample was collected in an adult psychiatric service, we could not include underage patients. Finally, we assessed parental educational levels in families studying fathers and mothers. New forms of family are increasingly emerging inside our society, like, for example, single-parent and homogenitorial families. More research is required to expand the analysis to these possibilities other than traditional and assess whether our findings are replicated.

Identifying the psychosocial factors that contribute to modulating some aspects of EDs is important to improving and tailoring the treatments offered. According to the present study’s findings, clinicians are recommended to consider family dynamics, parental educational level, and the role of features of perfectionism. Considering these psychosocial factors might lead clinicians to provide a more refined approach to individuals with EDs and their families.

## Data availability statement

The raw data supporting the conclusions of this article will be made available by the authors, without undue reservation.

## Ethics statement

The studies involving humans were approved by Comitato etico Città Della Salute e della Scienza di Torino. The studies were conducted in accordance with the local legislation and institutional requirements. The participants provided their written informed consent to participate in this study.

## Author contributions

FB: Conceptualization, Data curation, Formal analysis, Investigation, Writing – original draft. MM: Conceptualization, Data curation, Formal analysis, Writing – review & editing. PL: Conceptualization, Data curation, Writing – original draft. FT: Conceptualization, Data curation, Writing – original draft. AM: Data curation, Formal analysis, Writing – review & editing. LA: Data curation, Formal analysis, Writing – original draft. GA: Conceptualization, Data curation, Formal analysis, Supervision, Writing – review & editing, Writing – original draft. MP: Conceptualization, Data curation, Investigation, Methodology, Writing – original draft, Writing – review & editing.
