# Supplementary material for: Role of parental educational level as psychosocial factor in a sample of inpatients with anorexia nervosa and bulimia nervosa
Source: Front Psychol. 2024 May 17;15:1408695. doi: 10.3389/fpsyg.2024.1408695 (PMC11140136; doi:10.3389/fpsyg.2024.1408695)
Supplement: Supplementary file 1 [file Data_Sheet_1.docx]

Table S1. Parents’ educational level (years of education) in the clinical sample.

|  | Mothers | | Fathers | |
| --- | --- | --- | --- | --- |
|  | **N** | **%** | **N** | **%** |
| Not answered | 18 | 7.4 | 25 | 10.3 |
| 5 years | 14 | 5.8 | 19 | 7.9 |
| 8 years | 38 | 15.7 | 44 | 18.2 |
| 11 years | 4 | 1.7 | 6 | 2.5 |
| 12 years | 12 | 5.0 | 9 | 3.7 |
| 13 years | 101 | 41.7 | 89 | 36.8 |
| > 13 years | 55 | 22.7 | 43 | 17.8 |

Legend: N = number of individuals; % = percentage.

Table S2. Diagnostic subgroups composition of the clinical sample.

|  | N | % |
| --- | --- | --- |
| AN-R | 152 | 62.8 |
| AN-BP | 64 | 26.4 |
| BN | 26 | 10.7 |

Legend: N = number of individuals; % = percentage; AN-R = Anorexia Nervosa Restricting subtype; AN-BP = Anorexia Nervosa binge-purging subtype; BN = Bulimia Nervosa.

Table S3. Marital status of the individuals in the clinical sample.

|  | EDs individuals with mothers of >13 years of education | | EDs individuals with mothers of <13 years of education | | χ² | p | EDs individuals with fathers of >13 years of education | | EDs individuals with fathers of <13 years of education | | χ² | p | EDs individuals with both parents of >13 years of education | | EDs individuals with at least one parent of <13 years of education | | χ² | p | |
| --- | --- | --- | --- | --- | --- | --- | --- | --- | --- | --- | --- | --- | --- | --- | --- | --- | --- | --- | --- |
|  | **N** | **%** | **N** | **%** | **8.128** | **.043** | **N** | **%** | **N** | **%** | **9.368** | **.025** | **N** | **%** | **N** | **%** | **9.481** | **.024** |  |
| Single | 131 | 84.0 | 64 | 74.4 |  |  | 112 | 83.0 | 80 | 77.7 |  |  | 105 | 84.7 | 86 | 75.4 |  |  |  |
| Engaged | 21 | 13.5 | 16 | 18.6 |  |  | 19 | 14.1 | 15 | 14.6 |  |  | 18 | 14.5 | 19 | 16.7 |  |  |  |
| Married/Cohabitant | 2 | 1.3 | 6 | 7.0 |  |  | 0 | .0 | 7 | 6.8 |  |  | 0 | .0 | 8 | 7.0 |  |  |  |
| Divorced | 2 | 1.3 | 0 | .0 |  |  | 1 | .7 | 1 | 1.0 |  |  | 1 | .8 | 1 | .9 |  |  |  |
| Not answered | 0 | .0 | 0 | .0 |  |  | 3 | 2.2 | 0 | .0 |  |  | 0 | .0 | 0 | .0 |  |  |  |

Legend: EDs = eating disorders; N = number of individuals; % = percentage.

Table S4. Chi-square comparison between Maternal PEL and Paternal PEL

|  | Fathers of <13 years of education | Fathers of ≥13 years of education | Tot | χ² | p |
| --- | --- | --- | --- | --- | --- |
| Mothers of <13 years of education | 79 (34%) | 7 (2.6%) | 86 | **133.327** | **<.001** |
| Mothers of ≥13 years of education | 24 (9.8%) | 128 (53,6%) | 152 |  |  |
| Tot | 103 | 135 | 238 |  |  |

Legend: PEL = Parental Educational Level, Missing: N=6.

Table S5. Linear regression model for Personal Standard

|  |  |  |  | 95% CI | |  |  |  |
| --- | --- | --- | --- | --- | --- | --- | --- | --- |
| Type | **Effect** | **Estimate** | ***SE*** | **Lower** | **Upper** | **β** | **t** | **p** |
| Indirect | PEL ⇒ Personal Standard ⇒ Dietary Restrictions | .609 | .203 | .212 | 1.006 | .605 | 3.008 | **.003** |
| Component | PEL ⇒ Personal Standard  Personal Standard ⇒ Dietary Restrictions | 2.582  .236 | .830  .019 | .948  .035 | 4.216  .109 | 2.582  .072 | 3.111  3.847 | **.002**  **< .001** |
| Direct | PEL ⇒ Dietary Restrictions | -.123 | .253 | -1.004 | -.006 | - .505 | -1.991 | **.048** |
| Total | PEL ⇒ Dietary Restrictions | .269 | .019 | .045 | .119 | .082 | 4.366 | **< .001** |

Legend: PEL = Parental Educational Level

*Statistical significance if p < .05.

Table S6. Linear regression model for Parental Criticism

|  |  |  | |  | 95% CI | |  |  |  |
| --- | --- | --- | --- | --- | --- | --- | --- | --- | --- |
| Type | **Effect** | **Estimate** | | ***SE*** | **Lower** | **Upper** | **β** | **t** | **p** |
| Indirect | PEL ⇒ Parental Criticism ⇒ Dietary Restrictions | | - .042 | .150 | -.337 | .252 | - .042 | -.282 | .778 |
| Component | PEL ⇒ Parental Criticism  Parental Criticism ⇒ Dietary Restrictions | -.148  .286 | | .522  .030 | -2.268  .081 | -.213  .199 | -1.240  .140 | -2.377  4.706 | **.018**  **< .001** |
| Direct | PEL ⇒ Dietary Restrictions | -.123 | | .253 | -1.004 | -.006 | - .505 | -1.991 | **.048** |
| Total | PEL ⇒ Dietary Restrictions | -.074 | | .249 | -.792 | .189 | - .302 | -1.210 | .228 |

Legend: PEL = Parental Educational Level

*Statistical significance if p < .05.
